# A Systematic Review and Meta-Analysis on the Safety of Vascular Endothelial Growth Factor (VEGF) Inhibitors for the Treatment of Retinopathy of Prematurity

**DOI:** 10.1371/journal.pone.0129383

**Published:** 2015-06-17

**Authors:** Laura Pertl, Gernot Steinwender, Christoph Mayer, Silke Hausberger, Eva-Maria Pöschl, Werner Wackernagel, Andreas Wedrich, Yosuf El-Shabrawi, Anton Haas

**Affiliations:** 1 Department of Ophthalmology, Medical University Graz, Auenbruggerplatz 4, 8036, Graz, Austria; 2 Department of Ophthalmology, Klagenfurt Hospital, Feschnigstraße 11, 9020, Klagenfurt am Wörthersee, Austria; University of Utah (Salt Lake City), UNITED STATES

## Abstract

**Introduction:**

Laser photocoagulation is the current gold standard treatment for proliferative retinopathy of prematurity (ROP). However, it permanently reduces the visual field and might induce myopia. Vascular endothelial growth factor (VEGF) inhibitors for the treatment of ROP may enable continuing vascularization of the retina, potentially allowing the preservation of the visual field. However, for their use in infants concern remains. This meta-analysis explores the safety of VEGF inhibitors.

**Methods:**

The Ovid Interface was used to perform a systematic review of the literature in the databases PubMed, EMBASE and the Cochrane Library.

**Results:**

This meta-analysis included 24 original reports (including 1.457 eyes) on VEGF inhibitor treatment for ROP. The trials were solely observational except for one randomized and two case-control studies. We estimated a 6-month risk of retreatment per eye of 2.8%, and a 6-month risk of ocular complication without the need of retreatment of 1.6% per eye. Systemic complications were only reported as isolated incidents.

**Discussion:**

VEGF inhibitors seem to be associated with low recurrence rates and ocular complication rates. They may have the benefit of potentially allowing the preservation of visual field and lower rates of myopia. Due to the lack of data, the risk of systemic side effects cannot be assessed.

## Introduction

Retinopathy of prematurity (ROP) is one of the major causes of childhood blindness in the industrialized world. It is caused by an abnormal growth of retinal blood vessels [1]. The incidence of ROP is constantly increasing as larger, more mature infants in countries, where expertise in neonatal and ophthalmologic care is nascent, survive to develop ROP and as more immature infants are surviving, which develop ROP despite excellent neonatal care [2]. Laser photocoagulation is the gold standard treatment for ROP. Although laser photocoagulation is successful in many cases, it might reduce the visual field [3] and contribute to the development of myopia [4]. Therefore, an alternative may be useful [5].

Vascular endothelial growth factor (VEGF) is recognized as an important factor in the vascularization of the retina and the development of ROP [1]. Angiogenesis of the retina commences at approximately 17 weeks postmenstrual age. At this stage the metabolic demands of the neural retina outpace the oxygen supplied by the choroid. This physiologic hypoxia causes VEGF secretion stimulating new vessel formation until vascular development is complete just prior to birth. In preterm infants, the sudden increase in oxygen saturation after birth causes a down-regulation of growth factors resulting in a disruption of retinal vascular development. This is followed by a phase in which the attenuated vasculature cannot supply enough oxygen to the developing retina [6]. This hypoxic state leads to a VEGF overexpression inducing pathologic and excessive neovascularization at the avascular junction [7,8].

Anti-VEGF agents are widely used to effectively treat diseases of neovascular origin in adult eyes. In ROP, they may stop or reduce pathologic neovascularization. The biggest advantage of anti-VEGF metabolites is that, in contrast to laser photocoagulation, the retina does not seem to be permanently damaged [9]. However, for the use of anti-VEGF agents in infants concern remains [10,11].

Systemic side effects are of particular interest, as preterm infants with proliferative ROP have a compromised blood-retinal barrier possibly allowing a large amount of VEGF inhibitors to enter the blood stream [12]. Intravitreal bevacizumab and to a lesser extent ranibizumab seem to suppress systemic VEGF and thus systemic side effects cannot be excluded [13–16]. In adults, it is still under debate whether intravitreal injections of VEGF inhibitors increase the risk of thrombotic events [17,18].

Laser photocoagulation remains the standard treatment for ROP. However, laser photocoagulation may destroy large areas of the retina [5]. Therefore an alternative treatment is of interest, especially for preterm infants with zone 1 ROP. Yet, the use of VEGF inhibitors raises issues on ocular and systemic side effects. There is still little evidence on the safety of intravitreal VEGF inhibitors for ROP treatment. This study addresses 7 years of published data on VEGF inhibitors safety in preterm infants. The specific aims of this study were to determine ocular and systemic complications after the use of VEGF inhibitors for the treatment of ROP.

## Methods

### Search history

From December 27^th^, 2014, until January 8^th^, 2015, we used an Ovid Interface to search for the following medical subject headings in the databases PubMed, EMBASE, and The Cochrane Library: “vascular endothelial growth factor” AND “Retinopathy of Prematurity”; “Bevacizumab” AND “Retinopathy of Prematurity”; “Ranibizumab” AND “Retinopathy of Prematurity”; “Aflibercept” AND “Retinopathy of Prematurity”; “Pegaptanib” AND “Retinopathy of Prematurity”. The reference lists of included studies were additionally scanned to identify potentially relevant reports. Two investigators (MD) performed the literature search and study selection.

All trials were included if they reported the use of VEGF inhibitors for retinopathy of prematurity stages 3 or 4. Studies were excluded, if VEGF Inhibitors were used for recurrences after initial treatment with laser photocoagulation or vitrectomy. All animal studies were excluded. Only studies in English language were included. All abstracts and conference proceedings that are not published in peer-reviewed journals were excluded. If VEGF inhibitors were used in combination with other treatment options, they were not included for quantitative analysis. Case reports, small case series (<6 eyes) and trials with selective reporting of cases were reported, but not included in the quantitative analysis.

The primary outcomes are ocular complications requiring retreatment, ocular complication without the need of retreatment and systemic complications. Secondary outcome is refractive error.

### Statistical methods

All statistical analyses were performed using MetaXL. We observed pronounced differences regarding the length of follow-up between the individual studies. This may lead to bias, because the risk of experiencing an event (e.g. retreatment, complication) will be *a priori* higher in a study with e.g. 24 months of follow-up than in a study with e.g. 3 months of follow-up. To minimize bias in the presence of this heterogeneity, we transformed the original endpoint proportions reported by the individual studies to (constant) rates. These rates were pooled using random-effects meta-analysis. Finally, the pooled estimate was transformed into a 6-month proportion of endpoint risk.” In detail, we first calculated the eyetime-at-risk in months for each study, defined as the product of the follow-up duration in months and the number of eyes that did not experience the respective endpoint, plus the product of the number of eyes that experienced the respective endpoint and half of the follow-up duration. This procedure assumes that events occurred on average at the temporal midpoint between baseline and study end. Then, we obtained study-specific endpoint rates by dividing the number of events experienced during follow-up by the eyetime-at-risk in months. For studies without any events during follow-up, we applied a continuity correction and set the number of events to 0.5. Between-study heterogeneity of endpoint risks were assessed using the I-squared statistic and Cochran’s Q. The study-specific endpoint rates were then meta-analyzed using a random effects model (“NumRate” Table function in MetaXL). Finally, to obtain 6-month risks of developing the endpoints of interest, we assumed a constant event rate and hence used the following formula:

$$risk=1-e^{-rate*t}$$,

where t is 6 months.

To derive a meta-analysis estimate for the refractive error, we calculated an arithmetic mean weighted by the number of patients in the individual studies.

## Results

### Included studies

Our search identified 665 records. Of those 70 original papers were found to be relevant for our study. From these 17 were case reports, 9 case series with <6 eyes, 2 case series with ≥6 eyes, 28 retrospective, 11 prospective non-randomized, 2 case-controls and 1 randomized trial. In 4 of these 70 trials, VEGF inhibitors were compared to laser photocoagulation. The other 66 trials were purely observational data.

**Fig 1:**
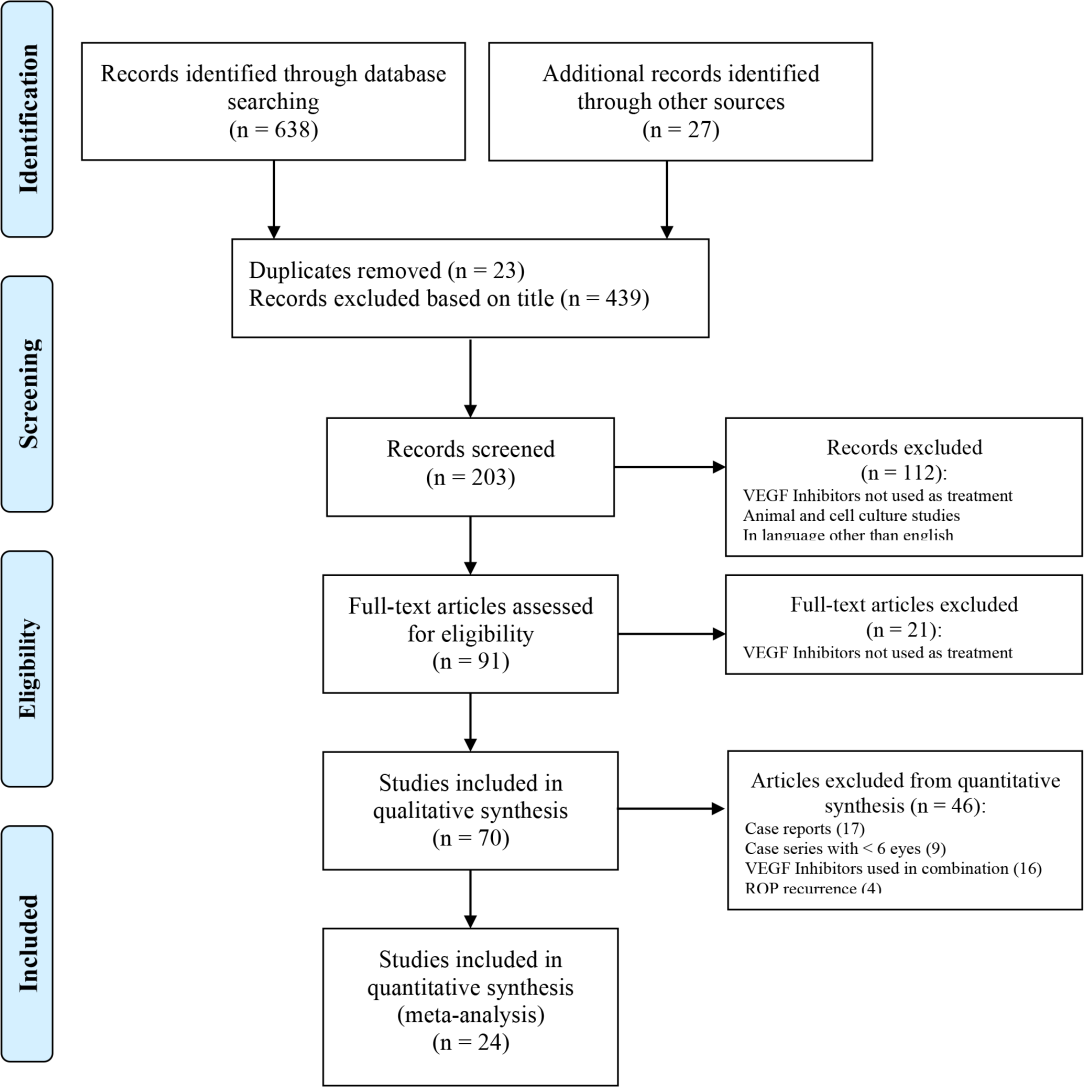
Flow chart of study exclusion. Adoption of the PRISMA flow diagram. It shows the number of trials identified, included and excluded, and reason for exclusions.

Case reports (17 trials), case series with less than six eyes (9 trials), trials investigating bevacizumab in combination with other treatment options (16 trials) and trials investigating the use of VEGF inhibitors for recurrences (4 trials) were excluded from quantitative analysis (see Fig 1).

24 studies with 1.457 eyes were included for quantitative analysis 21 (see Tables 1 and 2 and S1 Table). Out of these 1.457 eyes, 1.121 eyes (596 patients) fitted our criteria (no combined treatment, no recurrence). Of these 24 studies, ranibizumab was used in two and aflibercept in one trial.

**Table 1:** Characteristics of included studies.

Study	Study Type	Number of eyes	Intervention	Follow-Up (months)**	Ocular complication requiring retreatment	Ocular complication without the need of retreatment
Bancalari, 2014 [19]	retrospective	24	Bevacizumab	12	8 (33.3%)	1 (4.2%)
Chen, 2014 [20]	retrospective	72	Ranibizumab Bevacizumab	12	0 (0%)	0 (0%)
Chen, 2014 [21]	retrospective	57*	Bevacizumab w/ or w/o Laser coagulation	24	-	-
Cheng, 2014 [22]	retrospective	13	Bevacizumab	-	1 (7.7%)	0 (0%)
Geloneck, 2014 [23]	Follow-Up of Mintz-Hittner (38)	211*	Bevacizumab Laser coagulation	30	-	-
Harder, 2014 [24]	retrospective	57	Bevacizumab	-	1 (1.8%)	0 (0%)
Henaine-Berra, 2014 [25]	prospective, non-randomized	46	Bevacizumab	-	0 (0%)	0 (0%)
Kuniyoshi, 2014 [26]	retrospective	8	Bevacizumab	-	2 (25%)	0 (0%)
Lepore, 2014 [27]	case-control	24*	Bevacizumab Laser coagulation	12	0 (0%)	0 (0%)
Moran, 2014 [28]	case-control	28*	Bevacizumab Laser coagulation	24	3 (21.4%)	0 (0%)
Salman, 2014 [29]	prospective, non-randomized	26	Aflibercept	12	1 (3.8%)	0 (0%)
Yetik, 2014 [30]	prospective, non-randomized	238	Bevacizumab	-	11 (4.6%)	0 (0%)
Castellanos, 2013 [31]	prospective, non-randomized	6	Ranibizumab	36	0 (0%)	0 (0%)
Harder, 2013 [32]	retrospective	49*	Bevacizumab Laser coagulation	36	0 (0%)	0 (0%)
Martinez-Castellanos, 2013 [33]	prospective, non-randomized	18*	Bevacizumab	60	0 (0%)	0 (0%)
Sahin, 2013 [34]	retrospective	30	Bevacizumab	-	2 (6.7%)	5 (16.7%)
Wu, 2013 [35]	retrospective, multi-center	162	Bevacizumab	-	19 (11.7%)	4 (2.5%)
Dani, 2012 [36]	prospective, non-randomized	7*	Bevacizumab w/ or w/o Laser coagulation	-	0 (0%)	0 (0%)
Harder, 2012 [37]	prospective, non-randomized	32*	Bevacizumab Laser coagulation	12	-	-
Axer-Siegel, 2011 [38]	retrospective	18*	Bevacizumab w/ or w/o Laser coagulation	-	1 (10%)	0 (0%)
Harder, 2011 [39]	retrospective	23	Bevacizumab	-	0 (0%)	0 (0%)
Mintz-Hittner, 2011 [40]	prospective, randomized	286*	Bevacizumab Laser coagulation	12	6 (4.3%)	-
Dorta, 2010 [41]	retrospective	12	Bevacizumab	-	0 (0%)	1 (8.3%)
Mintz-Hittner, 2008 [42]	retrospective	22	Bevacizumab	-	0 (0%)	0 (0%)

Studies are sorted by date of publication.

*Not all patients included for quantitative analysis, as only selected patient groups fitted our criteria (no combined treatment, no recurrences).

**The follow-up period is only reported if follow-up was a defined period of time.

**Table 2:** Characteristics of included studies for refractive error.

Study	Study Type	Number of eyes	Intervention	Follow-Up (months)*	Refractive Error	Refractive Error
					Sphere	Cylinder
Chen, 2014 [21]	retrospective	40	Bevacizumab	24	0.98	2.23
		17	Bevacizumab with laser coagulation	24	-2.4	2.23
Geloneck, 2014 [23]	Follow-Up of	110	Bevacizumab	30	-1,02	
	Mintz-Hittner (38)	101	Laser coagulation	30	-6,73	
Harder, 2013 [32]	retrospective	23	Bevacizumab	12	0	-1
		26	Laser coagulation	12	-5.5	1.5
Harder, 2012 [37]	prospective,	12	Bevacizumab	-	0.635	1.015
	non-randomized	20	Laser coagulation	-	-5.225	1.69
Axer-Siegel, 2011 [38]	retrospective	10	Bevacizumab	-	0.5	0.5
		8	Bevacizumab with laser coagulation	-	-7.25	0
Mintz-Hittner, 2011 [40]	prospective,	140	Bevacizumab	12	0.99	-
	randomized	146	Laser coagulation	12	7.01	-

Studies are sorted by date of publication.

*The follow-up period is only reported if follow-up was a defined period of time.

All other trials investigated bevacizumab. The dosage for bevacizumab was 0.625mg in twelve, 0.25mg in one, 0.375mg in four, 0.5mg in one, 0.75mg in one and 1.25mg in two trials. The two trials investigating ranibizumab used 0.25mg and the trial investigating aflibercept used 1.0mg.

### Ocular complications

Ocular complications could be calculated for 882 eyes. Of these 55 eyes (6.2%) developed an ocular complication requiring retreatment. Using random effects meta-analysis, we estimated the 6-month risk of developing an ocular complication requiring retreatment at 2.8%. The reasons for retreatment were recurrent neovascularization in 32 cases (58.2%), retinal hemorrhage in 10 cases (18.2%), retinal detachment in 9 cases (16.4%), partial retinal detachment in 1 case (1.8%), macular dragging in 2 cases (3.6%) and persistent plus disease in 1 case (1.8%).

Ocular complications without the need of retreatment occurred in 11 (1.2%) out of 882 eyes. The 6-month risk of this endpoint was estimated at 1.6% per eye. The ocular complications were retinal hemorrhage spontaneously regressing in 8 cases (72.7%), cataract in 1 case (9.1%), mild macular traction in 1 case (9.1%) and exotropia in 1 case (9.1%). There was no correlation of ocular complication rate and dosage.

### Systemic complications

Systemic complications were investigated in 585 patients. Of these 8 patients (1.4%) reported systemic complications after intravitreal injections. However, in none of the cases the treatment with VEGF inhibitors was considered to be the cause of the complication.

In the only randomized study by Mintz-Hittner [40], five infants died in the bevacizumab group compared to two infants in the laser photocoagulation group. The five patients receiving intravitreal bevacizumab died of asphyxia (two infants, 16.3 and 12.3 weeks after intravitreal injection of bevacizumab or IVB), respiratory failure (two infants, 4.8 and 13 weeks after IVB) and adherence to do-not-resuscitate order (one infant, 0.4 weeks after IVB). The infants of the control group died of sepsis (one infant, 4.8 weeks after laser photocoagulation) and respiratory failure (one infant, 4.2 weeks after laser photocoagulation). In a case-control study, there was a report of an infant, who died after intravitreal injection [27]. In one study, one infant showed delay in growth, pulmonary dysplasia and intraventricular hemorrhage [33]. The vascular activity in the contralateral eye decreased after unilateral injection of bevacizumab in one patient [34].

The trials investigating ranibizumab and aflibercept [20,29,31] did not report any systemic complications.

### Studies, not included in quantitative analysis

In the studies, which were not included in this papers quantitative analysis, the most frequent reported ocular complication was retinal detachment requiring treatment after intravitreal injection of VEGF inhibitors [43–50]. There was one case report of spontaneous reattachment [51]. Especially important are the reports of initial regression with delayed recurrent neovascularization after bevacizumab (with or without laser photocoagulation), which occurred up to 19 weeks after intravitreal injection [52–57]. Other serious side effects included acute contraction of proliferative membrane [44,58–59], vascular sheathing [57], choroidal ischemia [60], optic atrophy [61], disc pallor [62], retinal break, RPE rupture [61] and choroidal rupture [63], which all occurred after bevacizumab combined with laser photocoagulation. Less severe side effects were cataract [51,57], increased IOP [51,64] and mild vitreous organization [65].

At the same time there are also many reports of VEGF inhibitor use in ROP without any ocular complications [66–78].

There were 27 trials with 592 patients not included in this papers quantitative analysis investigating systemic complications. In 25 trials no systemic complications were reported [43–44,47,49–50,57,59,62,64–68,74–85]. There was one report of short-term raised liver enzymes after bevacizumab injection [61] and one case report showing reduced vascular activity in the contralateral eye [5].

## Discussion

In this meta-analysis, retreatment was required in 2.8% of cases within 6 months and ocular complication rates without the need of retreatment were 1.6% per eye. Systemic complications were only reported as isolated incidents.

Laser treatment has proven useful to reduce progression of ROP. However, visual outcomes especially for zone 1 ROP are poor [86] and progression requiring retreatment occurs in approximately 11–20% of eyes despite adequate peripheral ablation [78,87–91]. Laser photocoagulation is associated with visual field defects, severe myopia, strabismus, glaucoma, cataract formation, corneal edema, and intraocular hemorrhage [86,92–95].

Intravitreal injection of VEGF inhibitors may be a useful alternative, especially for zone 1 ROP, as it allows continuing vascularization of the retina. Thus it may reduce visual field defects.

Further, VEGF inhibitors may reduce the incidence of severe myopia. The mean refractive error was always lower after intravitreal VEGF inhibitors if compared to laser photocoagulation (see Table 2). This difference is speculated to be a result of the minimal or absent development of the anterior segment of the retina after laser photocoagulation [23].

VEGF inhibitors might also be useful in severe cases of ROP for salvage treatment. Injection of VEGF inhibitors may reduce vascularization and thus allow laser treatment [48,73,78,84–85]. It might also improve the outcome after vitrectomy [77,96] and lower recurrences if combined with laser photocoagulation [82].

There are, however, many open questions. The biggest is on safety of VEGF inhibitors for preterm infants. Retreatment rates of ROP (e.g. retinal detachment or neovascularization requiring retreatment) were low in our meta-analysis. Similarly ocular side effects, which did not require retreatment, such as cataract, IOP increase and retinal hemorrhage were low. In the only randomized study comparing both modalities by Mintz-Hittner [40], the recurrence rate was higher in laser photocoagulation (31.7% respectively 5.9%).

A definite disadvantage of VEGF inhibitors is the possibility of systemic side effects. In our meta-analysis there are only few reported cases. Systemic side effects are however difficult to assess, as infants with ROP develop neurologic and other developmental disorders more often than other infants [96]. In fact in only selected studies [28,33,50] pediatric assessment and/or an MRI brain scan were performed specifically to evaluate any abnormalities. No systemic complications were found in these cases. However the number needed to definitely determine an increase in mortality after bevacizumab injection compared to laser treatment for ROP is estimated at 2800 infants [40].

**Fig 2:**
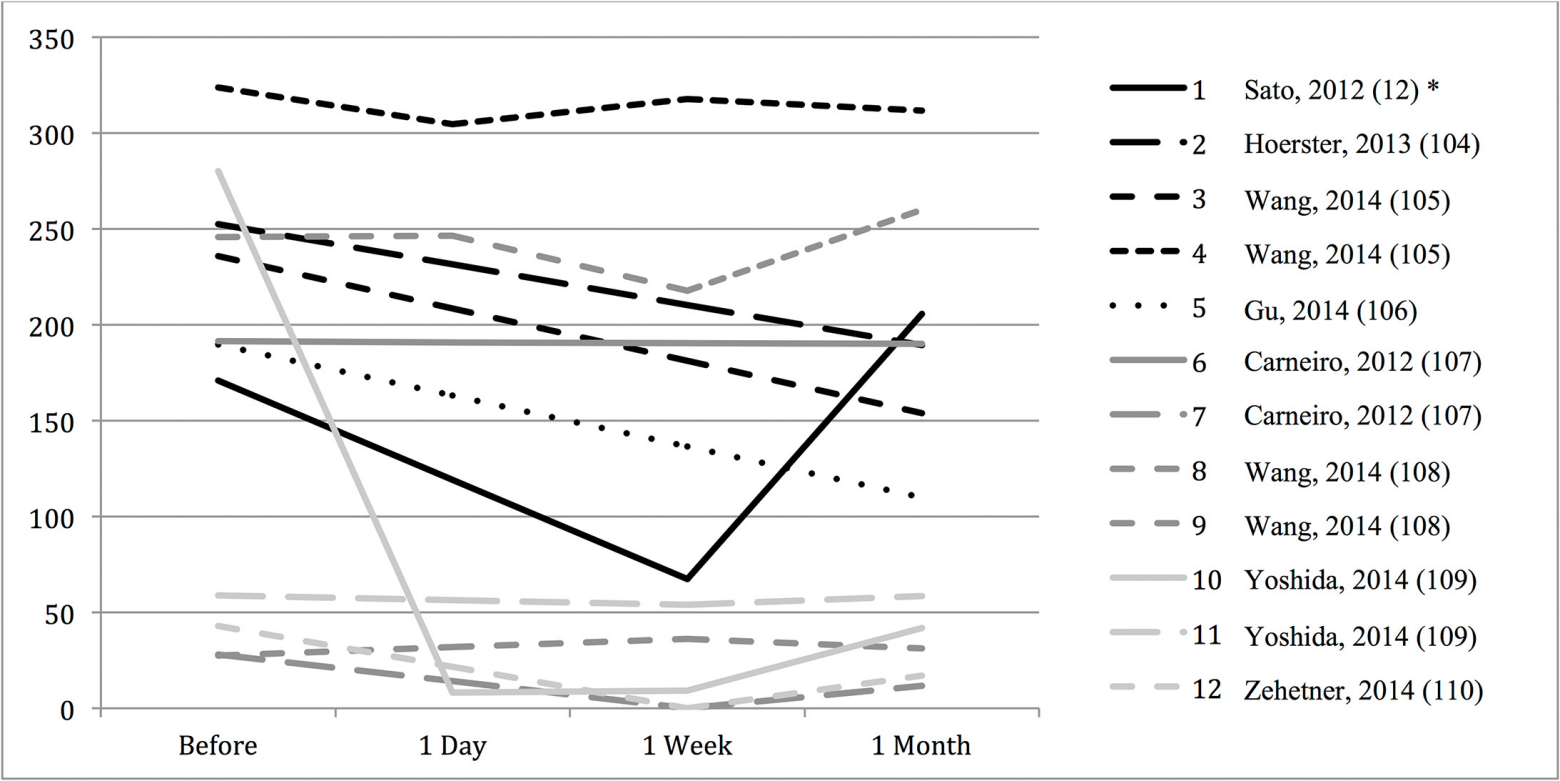
Systemic VEGF suppression after VEGF inhibitors. Research papers investigating systemic levels of VEGF after intravitreal injection of VEGF inhibitors over a defined period of time. *1 = infants with retinopathy of prematurity, unilateral injection of bevacizumab (14); 2 = infants with retinopathy of prematurity, unilateral injection of ranibizumab (105); 3 = patients with age-related macular degeneration (AMD), unilateral injection of bevacizumab (106); 4 = patients with AMD, bilateral injection of bevacizumab (106); 5 = patients with AMD, unilateral injection of ranibizumab (107); 6 = patients with AMD, unilateral injection of bevacizumab (108); 7 = patients with AMD, unilateral injection of ranibizumab (108); 8 = patients with AMD, unilateral injection of aflibercept (109); 9 = patients with AMD, unilateral injection of ranibizumab (109); 10 = patients with AMD, unilateral injection of ranibizumab (110); 11 = patients with AMD, unilateral injection of aflibercept (110); 12 = patients with AMD, unilateral injection of ranibizumab (111). ** y-axis in pg/ml.

In adults, systemic complications of intravitreal VEGF inhibitors are still under debate. Some studies showed an increase in mortality, while others did not (see Table 3). After intravitreal injection, VEGF inhibitors are found in the systemic circulation and systemic VEGF levels decrease [97–103]. Therefore concern remains about systemic toxicity of intravitreal VEGF inhibitors in infants (see Fig 2).

**Table 3:** Adverse events after anti-VEGF treatment in adults in relative risk.

Study	Study Type	VEGF inhibitor	Disorder	Deaths	Thromboembolic adverse events	Non-ocular hemorrhage
Thulliez, 2014 [18]	Meta-analysis	Ranibizumab	DME RVO AMD	-	1.14	-
Solomon, 2014 [104]	Meta-analysis	Pegaptanib	AMD	1.12	0.63**	-
	Meta-analysis	Ranibizumab	AMD	0.82	1.14	1.64
	Meta-analysis***	Bevacizumab vs Ranibizumab	AMD	1.28 (1.12)	1.07 (1.13)	-
Cheng, 2012 [105]	Meta-analysis	Ranibizumab Bevacizumab Pegaptanib	AMD DME RVO	0.68*	0.87	-
RESTORE 2014 [106]	Trial	Ranibizumab	DME		0.69	1.23
CATT, 2012 [107]	Trial	Bevacizumab vs Ranibizumab	AMD	1.7	1.11	-
ABC, 2010 [108]	Trial	Bevacizumab	AMD	1.02	1.02	-
FOCUS, 2008 [109]	Trial	Ranibizumab	AMD	1.87	0.86**	-
Marina, 2006 [110]	Trial	Ranibizumab	AMD	0.91	1.48	1.68

Studies are sorted by date of publication.

*Only vascular deaths were reported.

**Only cardiovascular events were reported.

***Events reported after 1 year (after 2 years).

Another possible disadvantage of anti-VEGF treatment are the reports of delayed recurrences, which occurred up to 19 weeks after injection. Thus, VEGF inhibitors require extensive, long-term follow-up [46].

To reduce complications, the lowest sufficient dosage should be used to treat ROP. At the moment, the recommended bevacizumab dosage for ROP infants is 0.625mg, which is half the adult dosage. However, the size-adjusted dose would be 0.4mg, which may be sufficient [111]. In our meta-analysis, there was no correlation of ocular complication rate and dosage. There is not enough evidence to support a recommended dosage.

In the trials using ranibizumab [20,31] no ocular and no systemic complications were reported. In the trial investigating aflibercept [29] one eye required retreatment, but no systemic complications were reported. Further research is needed on the comparison of safety and efficacy between bevacizumab, ranibizumab and aflibercept.

This is a meta-analysis of mostly observational data. Therefore, our data need to be interpreted in the sense that most of the evidence underlying our estimates stems from non-randomized trials. So far, there is only one randomized study by Mintz-Hittner [40]. Given that preterm infants are an especially vulnerable study group, this might not change in the near future. This paper explores the complications with the currently available literature.

There is a large heterogeneity in the studies included in this meta-analysis (see Table 1). Therefore we did not simply pool results, but used a random effects analysis to correct for the heterogeneity in the event rates. These differences in reported complication rates are probably not due to differences in the respective population but due to differences in reporting criteria. The endpoints studied in this meta-analysis were rarely clearly defined in the original papers and thus the criteria for reporting complications may differ greatly between studies.

The authors have no financial disclosure.

The data published so far suggest that intravitreal injection of VEGF inhibitors are a safe alternative for ROP treatment. The risk of systemic side effects cannot be fully assessed.

## Conclusion

VEGF inhibitors seem to be associated with low recurrence rates and ocular complication rates. They may have the benefit of potentially allowing the preservation of visual field and lower rates of myopia. However concern remains, especially about possible systemic complications. There is a dire need for larger and randomized trials on the safety of VEGF Inhibitors for the treatment of ROP.

## Supporting Information

S1 PRISMA Checklist Adoption of the PRISMA 2009 checklist for reporting of systematic reviews and meta-analyses. (TIF)

S1 Table Risk of bias of included studies. Studies are sorted by date of publication. Level of evidence as recommended by the Oxford Centre for Evidence-based Medicine. *Observational Studies.
(TIF)
