# Supplementary figures and images for: A Systematic Review and Meta-Analysis on the Safety of Vascular Endothelial Growth Factor (VEGF) Inhibitors for the Treatment of Retinopathy of Prematurity
Source: PLoS One. 2015 Jun 17;10(6):e0129383. doi: 10.1371/journal.pone.0129383 (PMC4470662; doi:10.1371/journal.pone.0129383)

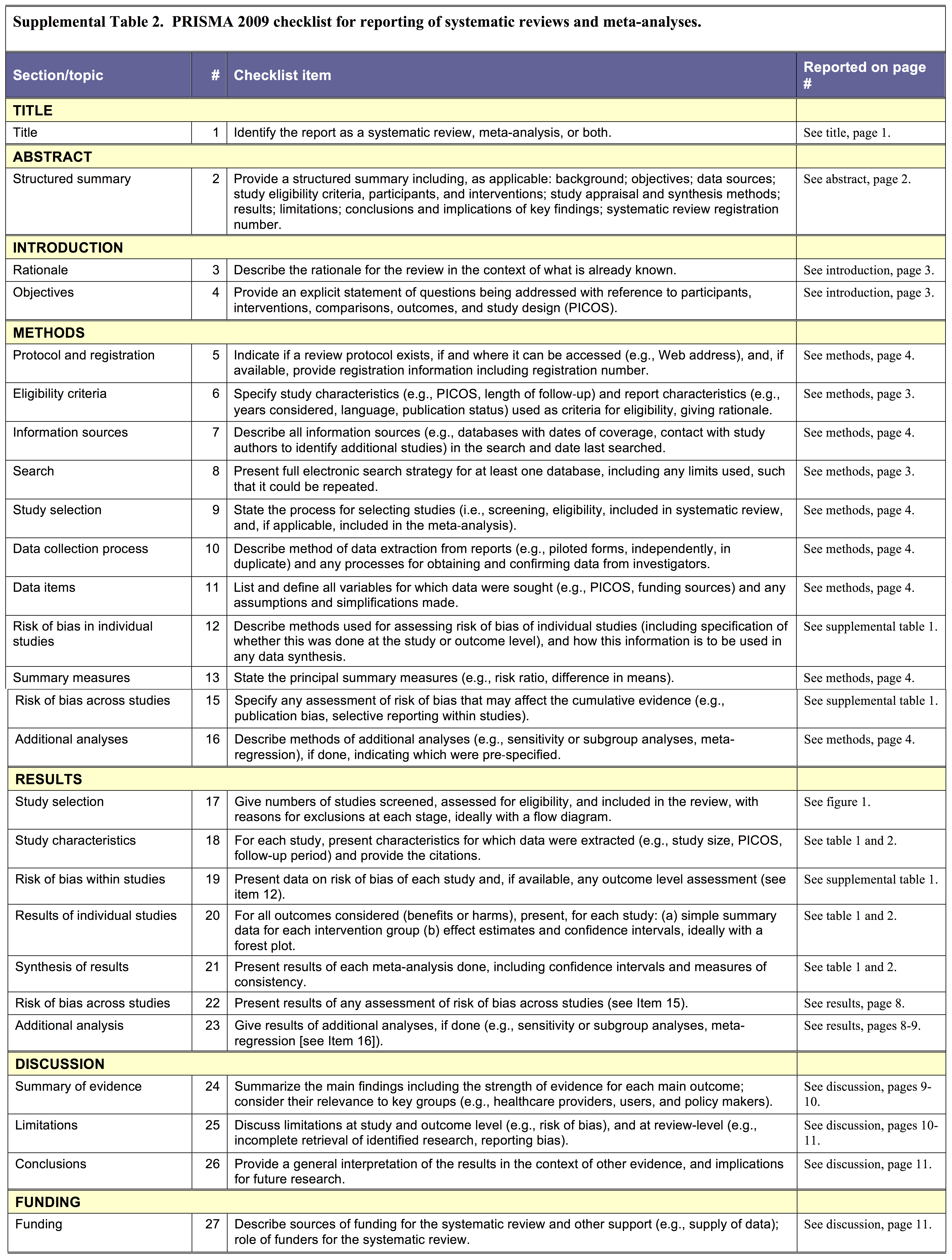

Supplement: S1 PRISMA Checklist — (TIF) [file pone.0129383.s001.tif]

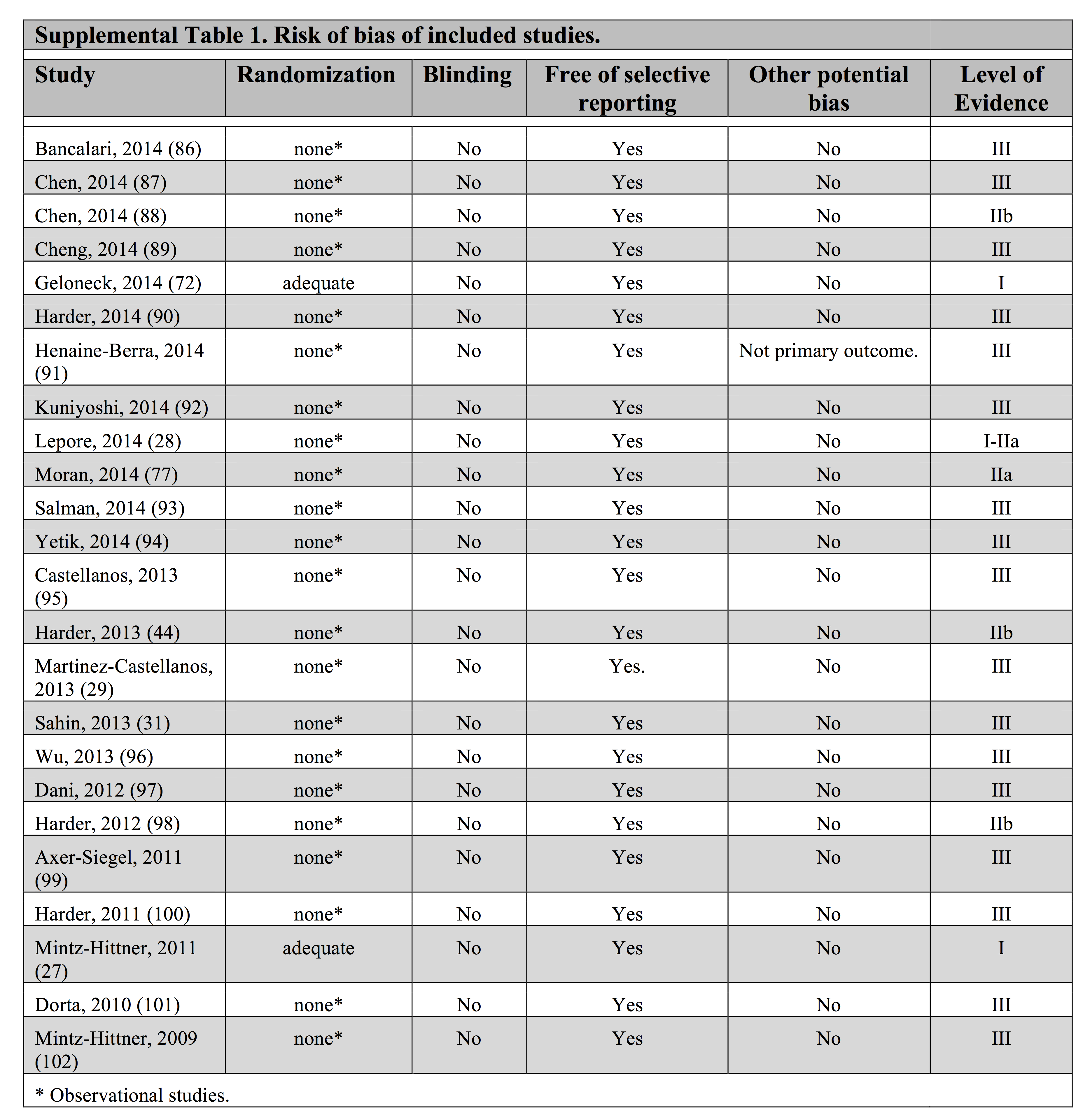

Supplement: S1 Table — Level of evidence as recommended by the Oxford Centre for Evidence-based Medicine. *Observational Studies. (TIF) [file pone.0129383.s002.tif]
